# Comparison of salivary beta-defensin-1 levels in patients with periodontitis before and after phase I periodontal therapy

**DOI:** 10.34172/japid.2024.002

**Published:** 2024-01-29

**Authors:** Somaye Ansari Moghadam, Sina Pishadast, Leila Gholami, Ebrahim Alijani, Alireza Ansari Moghadam, Mahdi Hadilou

**Affiliations:** ^1^Department of Periodontology, Oral and Dental Disease Research Center, Zahedan University of Medical Sciences, Zahedan, Iran; ^2^Oral and Dental Disease Research Center, Zahedan University of Medical Sciences, Zahedan, Iran; ^3^Department of Oral Biological and Medical Sciences, Faculty of Dentistry, University of British Columbia, Vancouver, Canada; ^4^Department of Clinical Immunology Research Center, Zahedan University of Medical Sciences, Zahedan, Iran; ^5^Department of Health Promotion Research Center, Zahedan University of Medical Sciences, Zahedan, Iran; ^6^Faculty of Dentistry, Tabriz University of Medical Sciences, Tabriz, Iran

**Keywords:** Beta-defensins, Chronic periodontitis, Enzyme-linked immunosorbent assay, Root planing

## Abstract

**Background.:**

This study compared human β-defensin 1 (*hBD-1*) salivary levels in patients with periodontitis before and after phase I periodontal therapy.

**Methods.:**

This controlled before-and-after study included 16 patients in the intervention group and 28 participants in the control group. Patients in the intervention group had stage 3 grade B periodontitis with no systemic diseases and had not taken any medications in the last six months. The control group included participants with healthy periodontium. Before and after phase I periodontal therapy, salivary samples were collected from the intervention group. ELISA was used to measure hBD-1 levels.

**Results.:**

Salivary levels of hBD-1 decreased after phase I periodontal treatment in periodontitis patients, approaching those in healthy individuals. However, this reduction was not statistically significant (*P*=0.389). In patients with a probing depth (PD) of at least 3 mm, salivary levels of hBD-1 decreased significantly (*P*=0.019) following the intervention. There was no significant correlation between changes in hBD-1 levels and clinical indices, such as clinical attachment loss (CAL), probing depth, or bleeding index (BI) (*P*˃0.05).

**Conclusion.:**

The current study demonstrated promising results concerning a probable link between hBD-1 and periodontitis. However, more research with sufficiently large sample sizes and more robust study designs is necessary.

## Introduction

 Periodontal diseases are caused by disturbances in the dynamic and complex equilibrium between bacterial plaque activity and human immunological responses, causing progressive damage to the periodontium, including periodontal ligaments, surrounding bone structures, gingival recession, pocket formation, and, eventually, tooth loss.^1,2^

 The human immune system includes innate and adaptive responses to any possible extrinsic or intrinsic threats. Antimicrobial peptides are one of the major components of the innate immune system that vary in size, amino acid arrangement, and specific structural properties.^3-5^ Human beta-defensins (hBDs) are cationic peptides synthesized by mucoepithelial cells in the gastrointestinal, respiratory, and urogenital tracts and salivary gland ducts.^6,7^ Since their production is regulated by microbial products, growth factors, and cytokines, they can also serve as indicators of existing inflammation.^8^

 Based on genomic targeting, the hBD family includes more than 20 defensins, of which types 1 to 4 are expressed in the gingival epithelium.^4,9^ According to the literature, hBD-1 has a broader range of antimicrobial activity than the other hBDs, and it is effective against both gram-negative and gram-positive bacteria.^10^ Unlike hBD-2 and -3 expression, which depends on bacterial stimulation, hBD-1 has a continuous expression in saliva and gingival crevicular fluid (GCF),^11^ and thus could be considered the frontline of immune system defense when encountered pathogens.

 According to recent reviews by Sulijaya et al^12^ and Dommisch and Jepsen,^4^ there are controversial findings and a lack of evidence regarding the possible mechanisms leading to a link between the periodontium’s condition and hBD-1 level and its direction. Both in vitro and in vivo studies confirm or refute this potential relationship,^3,13-16^ implying that more extensive studies with robust designs are needed.

 Since it reduces and alleviates inflammation and is non-invasive, scaling and root planing (SRP) are recommended as a significant part of the first phase of periodontal treatments before decision-making and planning for periodontal surgeries. This phase is considered the gold standard in periodontitis treatment.^17^

 To our knowledge, no study has evaluated the effect of phase I periodontal therapy on hBD-1 salivary levels in patients with periodontitis. Despite the site-specific nature of periodontitis, the accumulation of hBD-1 secreted from diseased sites in saliva could aid in the timely determination of the overall condition of the periodontium, diagnosis, severity, prognosis, and treatment of periodontitis, as well as revealing the role it might have as an indicator of ongoing inflammation. Therefore, the current study compared hBD-1 salivary levels in periodontitis patients before and after phase I periodontal therapy.

 The present study’s null hypotheses were as follows: (1) There was no difference in hBD-1 levels after the phase I periodontal therapy. (2) There would be no correlation between hBD-1 and periodontal indices, including clinical attachment loss (CAL), probing depth (PD), and bleeding index (BI).

## Methods

###  Study design and ethical considerations

**Figure 1:**
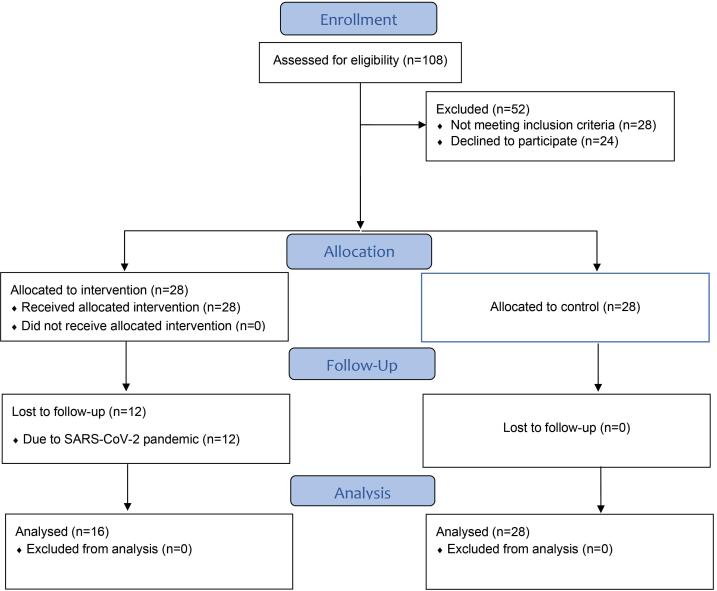
Flowchart of the study protocol

 From November 2019 to November 2020, patients referred to the Periodontics Department, Faculty of Dentistry, Zahedan University of Medical Sciences (Persian ethnicity) were enrolled in this controlled before-and-after study. The Zahedan University of Medical Sciences Ethics Committee approved the study protocol (IR.ZAUMS.REC.1398.256). Before beginning the study, the stages were thoroughly explained to the patients, and written informed consent was obtained. Patients could also quit whenever they preferred. The Consolidated Standards of Reporting Trials (CONSORT) were used to design, analyze, and interpret this study (Figure 1).

###  Selection criteria

 Inclusion criteria:

- Systemically healthy patients
- Patients with stage 3 grade B periodontitis

Exclusion criteria:

- Women with pregnancy
- Patients with dental caries
- On antibiotics or anti-inflammatory medications in the past six months
- Smoking
- Intention to discontinue treatment

###  Sample size calculation

 A power analysis was performed using G*Power V3.1 software to determine a suitable sample size with sufficient power to detect any potential difference, assuming an α = 0.05, β = 0.30, and mean values from Costa et al.^18^ As a result, a sample size of 28 patients in each group was determined.

###  Intervention procedures

 An experienced periodontist diagnosed patients with stage 3 grade B periodontitis based on the criteria introduced in the “consensus report of workgroup 2 of the 2017 world workshop on the classification of periodontal and peri-implant diseases and conditions”^19^ and performed phase I periodontal treatments, including oral health education and SRP. Repairing defective restorations and managing the carious lesions (also the parts of phase I periodontal therapy) were addressed by excluding patients with dental caries. Salivary samples were taken from the treatment group before and three months after the phase I periodontal treatment.

 At the end of the study, salivary samples from healthy individuals were collected simultaneously with the treatment group. Participants were asked not to eat, drink, or brush their teeth 90 minutes before sampling. The samples were collected between 9 and 11 a.m. The spitting method was used to collect non-stimulatory saliva from both intervention and control groups. After collecting salivary samples in 14-mL Falcon tubes, the tubes were transferred to the Immunology Laboratory of Zahedan Medical School while maintaining the cold chain (-70 °C temperature) until the tests were performed. An experienced laboratory technician used an ELISA (enzyme-linked immunosorbent assay) test to measure hBD-1 salivary levels according to the manufacturer’s instructions. Furthermore, clinical indices, including CAL, PD, and BI, were measured after treatment to evaluate the quality of treatments and investigate the correlation between changes in those indices and hBD-1 levels.

###  Quantification of salivary beta-defensin 1

 The superficial layers of saliva were instantly separated and stored at -70 °C after centrifuging salivary samples (at 2500 rpm for 10 minutes). hBD-1 concentration was measured using an ELISA kit (Lot: E2019010010, Hangzhou EASTBIOPHARM CO., LTD, China) according to the manufacturer’s instructions. In brief, the standard wells received 50 μL of standard solution + 50 μL of streptomycin-HRP (Horseradish Peroxidase), while the sample wells received 40 μL of the sample + 10 μL of DEFB1 (human beta-defensin-1 antibodies) + 50 μL of streptomycin-HRP. After 60 minutes of incubation at 37 ºC and washing, 50 μL of chromogen solution A was added to the wells, followed by 50 μL of chromogen solution B. After 10 minutes of incubation at 37ºC and adding a stop solution, the reaction was blocked, and the optical density (absorbance) of each well was measured with an ELISA reader at 450 nm wavelength. The lowest measurable limit of hBD-1 was 2.31 pg/mL. The standard curve’s linear regression equation was computed using the standards’ concentrations and their associated optical density values. The concentration of hBD-1 was then computed according to the corresponding optical densities.

###  Data analysis

 The primary outcome variable was hBD-1 salivary concentration. It was presented as means and standard deviations (mean ± SD) in nanograms/milliliters (ng/mL). The paired sample *t* test and ANOVA parametric tests were performed using the Kolmogorov–Smirnov test. Pearson’s correlation was used to assess the correlation of CAL, PD, and BI alterations with hBD-1 levels. The data were analyzed using SPSS 16, and a 0.05 significance level was considered statistically significant.

## Results

 We enrolled 28 patients in the treatment group, but due to the SARS-CoV-2 pandemic, only 16 (57.1%) returned for follow-ups. Finally, the treatment group included 16 patients, and the control group included 28 participants (Figure 1). The treatment group included 5 males and 11 females with a mean age of 36.62 ± 6.17 years, and the control group included 13 males and 15 females with a mean age of 39.21 ± 4.10 years.

 According to Table 1, all periodontal parameters improved significantly after phase I periodontal therapy in both subgroups based on the PD (*P* < 0.05).

**Table 1:** Phase I periodontal therapy and alterations in clinical indices

**Parameters**	**Probing depth**	**Before**	**After**	***P*** value*
CAL	< 3 mm	2.89 ± 0.90 mm	2.35 ± 0.73 mm	< 0.001
	≥ 3 mm	3.93 ± 1.02 mm	3.25 ± 0.80 mm	0.001
BI	< 3 mm	90.88 ± 12.12%	19.55 ± 13.33%	< 0.001
	≥ 3 mm	88.00 ± 16.12%	25.14 ± 12.58%	< 0.001
PD	< 3 mm	2.40 ± 0.45 mm	1.84 ± 0.43 mm	< 0.001
	≥ 3 mm	3.55 ± 0.39 mm	2.92 ± 0.46 mm	< 0.001

^*^Paired t-test; BI: bleeding index; CAL: clinical attachment loss; PD: probing depth.

 The salivary hBD-1 level in healthy individuals was 401.00 ± 234.73 (ng/mL). There was no significant difference in hBD-1 levels before (499.35 ± 199.11) and after (419.73 ± 250.78) the phase I periodontal therapy in patients with periodontitis (*P* = 0.389, by ANOVA). The hBD-1 level decreased significantly in participants with PDs of at least 3 mm (*P* = 0.019), but no difference was observed in those with PDs lower than 3 mm after the intervention (*P* = 0.405) (Table 2). According to Table 3, there was no correlation between the hBD-1 level and CAL, PD, and BI changes (*P*˃0.05).

**Table 2:** hBD-1 levels in patients with periodontitis before/after intervention

**Clinical index**	**PD**	**Before (ng/mL)**	**After (ng/mL)**	***P*** value*
PD	< 3 mm	442.01 ± 166.22	523.53 ± 271.37	0.405
	≥ 3 mm	573.07 ± 225.84	286.29 ± 148.66	0.019

^*^Paired t-test; PD: probing depth.

**Table 3:** Correlation of hBD-1 with CAL, PD, and BI

**Clinical index**	**Pearson’s correlation coefficient**	***P*** value*
CAL	0.314	0.236
PD	0.227	0.398
BI	-0.044	0.873

^*^Pearson’s correlation; PD: probing depth; CAL: clinical attachment loss; BI: bleeding index.

## Discussion

 Determining the diagnosis, prognosis, and treatment type for periodontal diseases based on biomarker levels, such as beta-defensins or other antimicrobial peptides, provides a more accurate method that allows clinicians to avoid making decisions based solely on clinical observations, which may be biased.

 Several in vitro and in vivo studies have examined the potential association between salivary hBD-1 concentrations and the severity of periodontal diseases. Given the positive role of phase I periodontal therapy (mostly SRP) in treating periodontitis^17^ and the existing debates about the differences in hBD-1 levels in patients with periodontitis versus those with healthy periodontium,^3,10,20,21^ this study aimed to evaluate the effect of phase I periodontal treatment on the salivary levels of hBD-1 in patients with periodontitis before and after phase I periodontal therapy.

 The hBDs exert antibacterial activity by permeabilizing the cellular membrane through forming pores, leaking small molecules, and destroying the integrated bacterial inner structure, eventually leading to their death.^22^ The electrostatic interaction between hBDs’ cationic structure and negatively polarized bacterial membrane causes this antimicrobial characteristic. hBDs can also neutralize the lipopolysaccharide on the membrane of the gram-negative bacteria.^9^ On the contrary, bacteria resist by changing their cell envelope structure, nesting in biofilms, cleaving the hBDs, or inhibiting their expression pathways.^23,24^

 According to Table 1, the CAL, PD, and BI indices significantly improved following phase I periodontal therapy, indicating that the treatment was of high quality. A reduction in hBD-1 salivary levels after treatment, close to that of the control group’s healthy periodontium, was observed, which was not statistically significant (*P*˃0.05), confirming the study’s null hypothesis. However, the reduction in hBD-1 levels was significant (*P* < 0.05) in those with PDs of at least 3 mm. Ultimately, the findings revealed no correlation between CAL, PD, and BI periodontal indices and hBD-1 levels (*P*˃0.05).

 Wang et al^16^ reported that hBD-1 mRNA expression was higher in patients with periodontitis than those with healthy periodontium. Additionally, its expression was higher than that of other hBDs.In another study, Vardar-Sengul et al^10^ discovered that hBD-1 gene expression was higher in chronic periodontitis cases than gingivitis and aggressive periodontitis. Other studies found no significant difference in hBD-1 values regarding periodontium health conditions.^3^

 On the contrary, Costa et al^18^ showed that the hBD-1 level in GCF was higher in chronic periodontitis than in healthy periodontium.Ebrahem^25^ found that the hBD-1,-2,-3 mRNA expression levels in GCF before and after nonsurgical periodontal therapies accompanied by an antibiotic regimen (100 mg doxycycline twice daily for 10 days) were higher after the intervention in patients with localized aggressive periodontitis. They concluded that the results were due to the inhibitory effect of regulatory cytokines secreted in inflamed tissues on hBD-1 secretion. Thus, after the amelioration of inflammation, the reduction in the cytokine secretion increases the hBD-1 expression to reach pre-disease levels.

 As previously stated, the potential association and its direction between hBD-1 levels and periodontal condition is still unclear. It has been suggested that polymorphisms of the hBD-1-related gene (DEFB1) and ethnicity may cause these inconsistencies, as they may be connected to the diversity in the amounts of hBD-1 expression and severity of periodontitis that an individual develops.^12^ One of the hypotheses regarding various expression patterns of hBD-1 is that the expression does not decrease in periodontitis. Instead, individuals who develop periodontitis genetically have a low secretion of hBD-1, which is why they are prone to periodontal disease.^26^

 The other, more detailed hypothesis is that bacterial colonization increases hBD expression, which limits bacterial growth. However, resistant bacteria survive and invade the inner layers of gingival tissues. The chemoattractant peptides then attract phagocytes and lymphocytes to the infection site and inhibit the secretion of antimicrobial peptides such as hBD-1.^27^

 The current study’s limitations include:

1. A low rate of patients returning for follow-ups (57%) after treatment due to the SARS-CoV-2 pandemic surge
2. Lowering the power of the study to a moderate level (0.60)
3. Possibly, the final sample did not provide sufficient power to show differences in hBD-1 levels.

Furthermore, including a narrow subgroup of periodontitis (stage 3 grade B) may not adequately represent severe periodontitis. This issue should be addressed in future studies with larger sample sizes that include broader stages of periodontitis.

## Conclusion

 Before-and-after studies are typically the first steps in evaluating new hypotheses. The current study found a promising link between hBD-1 and periodontitis. Therefore, more research into this potential relationship is needed.

## Acknowledgments

 This controlled before-after study was part of a thesis conducted from November 2019 to November 2020.

## Competing Interests

 The authors declare that they have no competing interests.

## Consent for Publication

 Not applicable.

## Data Availability Statement

 The datasets used and/or analyzed during the current study are available from the corresponding author upon reasonable request.

## Ethical Approval

 The study protocol was approved by the Ethics Committee of Zahedan University of Medical Sciences (IR.ZAUMS.REC.1398.256).

## Funding

 The authors received no financial support for the research, authorship, and/or publication of this article.
